# A High-Performance Strain Sensor for the Detection of Human Motion and Subtle Strain Based on Liquid Metal Microwire

**DOI:** 10.3390/nano14020231

**Published:** 2024-01-21

**Authors:** He Zhu, Zheng Sun, Xin Wang, Hong Xia

**Affiliations:** 1 State Key Laboratory of Integrated Optoelectronics, College of Electronic Science and Engineering, Jilin University, 2699 Qianjin Street, Changchun 130012, China; 2 Department of Rheumatology, the First Hospital of Jilin University, Changchun 130012, China; wangxin123@jlu.edu.cn

**Keywords:** liquid metal nanoparticles, strain sensor, microwire, laser direct writing

## Abstract

Flexible strain sensors have a wide range of applications, such as human motion monitoring, wearable electronic devices, and human–computer interactions, due to their good conformability and sensitive deformation detection. To overcome the internal stress problem of solid sensing materials during deformation and prepare small-sized flexible strain sensors, it is necessary to choose a more suitable sensing material and preparation technology. We report a simple and high-performance flexible strain sensor based on liquid metal nanoparticles (LMNPs) on a polyimide substrate. The LMNPs were assembled using the femtosecond laser direct writing technology to form liquid metal microwires. A wearable strain sensor from the liquid metal microwire was fabricated with an excellent gauge factor of up to 76.18, a good linearity in a wide sensing range, and a fast response/recovery time of 159 ms/120 ms. Due to these extraordinary strain sensing performances, the strain sensor can monitor facial expressions in real time and detect vocal cord vibrations for speech recognition.

## 1. Introduction

Strain sensors play crucial roles in various fields, including healthcare, robotics, and human–computer interaction. These sensors enable the detection and measurement of mechanical deformations, providing valuable insights into human motion and subtle strain analysis [1,2]. Traditional strain sensors are mainly composed of rigid matter, having the disadvantages of high cost and poor ductility [3]. During the movement process, the rigidity causes discomfort and the instability of the sensing signal. Flexible strain sensors can meet all the sensing functions of the traditional strain sensors [4]. In addition, they have the advantages of low cost, good ductility, and good biocompatibility and are widely used in human motion monitoring, wearable electronic devices and electronic skin [5,6]. Therefore, the strain sensors are changing from rigid to flexible, which is the next development trend [7,8].

The flexible strain sensor is composed of a flexible substrate and a conductive active material. In recent years, various flexible substrates with excellent performance have been developed, such as platinum catalyzed silica gel and polymethylsiloxane [9], polyurethane (PU) [10,11]. These flexible substrate materials can be applied to various types of flexible strain sensors due to their good scalability and biocompatibility, meeting the needs of the development of flexible strain sensors towards miniaturization, integration, and intelligence. The conductive materials of the flexible strain sensors have also been researched, and a variety of new nano conductive materials have been developed, such as carbon nanotubes [12,13], metal nanowire, graphene [14,15], and other solid conductive materials [16].

Although a flexible strain sensor made of solid conductive material has good sensitivity, the solid conductive material and the flexible substrate will generate large internal stress or friction from the difference in the intrinsic deformation parameters. Hysteresis is generated [17] as well as the loss of the solid conductive material, which will affect the service life of the flexible strain sensor [18]. If this kind of flexible strain sensor deforms excessively, it may cause irreversible damage to the solid conductive material, making the sensor completely invalid, which limits the application of the sensors.

In addition, liquid metal [19], ionic liquid [20], reduced graphene oxide solution, and other liquid conductive materials [21] have obtained more attention. Due to the natural ductility and fluidity of the liquid itself, it not only adapts to a relatively large range of deformation but also reduces the friction between the conductive material and the flexible substrate. However, it is a common approach to prepare the liquid channels, inject liquid conductive materials into them and seal them [22]. The preparation process of many liquid flexible strain sensors is cumbersome, and the preparation efficiency is low. Liquid metallic materials, especially eutectic gallium indium (EGaIn, 75 wt% Ga and 25 wt% In), which remain liquid at room temperature and even at low temperatures, and at the same time have the advantages of good electrical conductivity and deformability, have been widely investigated in the field of flexible strain sensors and show promising applications.

Compared with macro sensors, micro sensors have many unique advantages, including low power consumption, wide applications, high wearing comfort, and portability, and they can easily realize array sensing and distributed measurement. At the same time, the continuous reduction in sensor size is conducive to the integration of different types of sensors and the realization of powerful sensing systems. Microsensors often involve complex fabrication processes and require advanced materials, which can contribute to a higher manufacturing cost. Additionally, a limited sensing range may not be suitable for applications requiring measurements over larger distances or volumes. The technology of realizing micro flexible strain sensor mainly includes lithography technology [23,24], printing electronic technology [25], and laser processing technology [26]. Lithography technology is widely used, which can enable high-precision and complex microstructure preparation. However, the preparation process steps are cumbersome, the selection of sensing materials is limited, and the flexible positioning and size selection of template dependence are limited. Printing electronic technology has a simple process flow and low cost and can quickly produce diversified microstructures. However, its processing accuracy is limited and a variety of toxic chemicals need to be added in the process of configuring the printing ink. In order to solve the abovementioned problems, this paper used femtosecond laser direct writing (FsLDW) technology with high processing accuracy, controllable computer programming, and no mask and toxic chemical reagents to prepare a micron wire flexible strain sensor with excellent performance and apply it to human health signal monitoring.

Here, the liquid metal nanoparticles (LMNPs) were prepared using the ultrasonic method, and then the FsLDW technology was used to induce the assembly and deposition of LMNPs to form microwires. A small-size micro wire flexible strain sensor was prepared. The LMNPs microwire is composed of a large number of nanoparticles burst-sintered. The sintered body connection makes the flexible strain sensor have better force sensing performance. The sensitivity of the sensor is as high as 76.18, the linear coefficient is 0.999, the response time is 159 ms, and the recovery time is 120 ms. The sensor can sense various dynamic strains in real time. The application of the micro linear strain sensor in human health monitoring is further explored. By installing the sensors in different parts of the human body, the monitoring of human health signals can be successfully performed. While the sensor is attached to a human face, the change and degree of change in expressions can be detected. In the same way, vocal cord vibrations can be detected for speech recognition when the sensor is attached to the outside of the throat.

## 2. Materials and Methods

### 2.1. Materials and Instruments

E-GaIn, eutectic gallium-indium alloys composed of 75.5% Ga and 24.5% In by weight (melting point 15.7 °C) and isopropyl alcohol were purchased from Sigma-Aldrich and used as received. The silver conductive paint was purchased from Beijing Xin Xing Bai Rui Technology Co., Ltd., Beijing, China.

Scanning electron microscope (SEM) images and Energy dispersive spectrometer (EDS) element distribution mapping images were characterized using a JEOL JSM-7500F, Tokyo, Japan. A thin layer of Pt was sputtered onto the sample for better SEM imaging.

### 2.2. Synthesis of LMNPs

**Figure 1:**
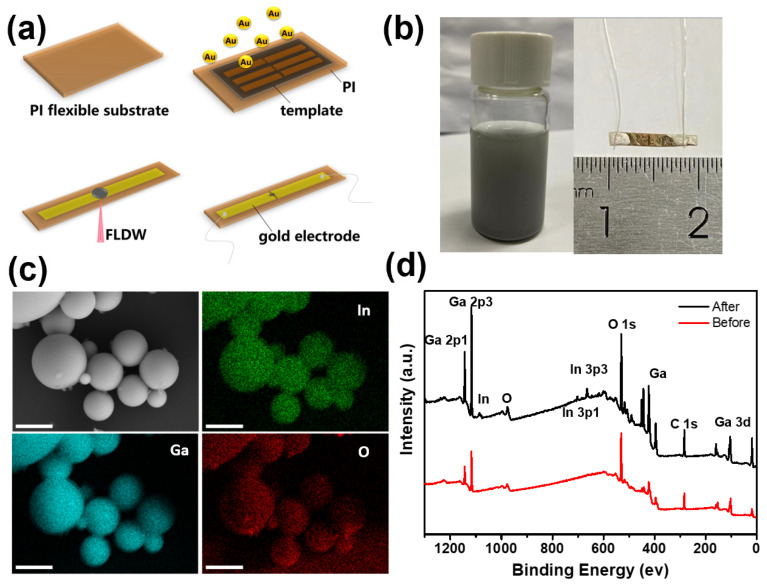
(**a**) Schematic illustration fabrication of a flexible strain sensor based on LMNPs by FsLDW. (**b**) The photo of LMNPs dissolved in 10 mL of isopropyl alcohol (**left**). The actual photo of the tested LM strain sensor (**right**). (**c**) The SEM and EDS mapping images of LMNPs. The scale bar is 500 nm. (**d**) The XPS spectra of LMNPs before and after laser processing.

First, 200 mg of EGaIn was placed in a glass vial filled with 30 mL isopropyl alcohol. The solution was sonicated using a probe sonicator (Scientz-ⅡD, Ningbo, China) with power 160 W for 30 min and cooled using an ice bath. Afterward, the large liquid metal droplets in the resulting suspension were removed by centrifugation with a rotating speed of 2000 rpm. Finally, the LMNPs suspension was dissolved in 10 mL of isopropyl alcohol for next-step fabrication, as shown in Figure 1b (left).

### 2.3. Fabrication Process and Test Instruments

The preparation process of the microwire flexible strain sensor from FsLDW of LMNPs is shown in Figure 1a. First, we chose a polyimide (PI) film with a thickness of 100 μm as flexible substrate, due to the flexibility, adhesion and thermal stability. The gold electrode was obtained through thermal evaporation on the surface of the PI substrate using a mask plate. The channel width between the electrodes was 100 μm. Then, the oxygen plasma was performed for two minutes to improve the wettability of the substrate. A drop of LMNPs dispersion was added dropwise at the area between the two electrodes. The concentration of LMNPs dispersion is about 10 mg/mL and the thickness of the liquid layer is about 1 mm. The laser scanned point by point according to the preset processing path. The fabrication system of the FsLDW referred to our previous work [27]. The femtosecond laser was emitted from a Ti:Sapphire femtosecond oscillator (Mai-Tai HP, Spectra-Physics 3960-X1BB, Milpitas, CA, USA) with a center wavelength of 800 nm, repetition frequency of 80 MHz, and pulse width of 120 fs. The laser power could be adjusted with a filter in the system. A high-precision electro-oscillatory scanning system (Sunnu Technology Ltd., Beijing, China) and a piezoelectric displacement stage (Power Integrations, Inc., San Jose, CA, USA) could be programmed to move the spot in the X-Y and Z directions. Integrations, Inc. (Exton, PA, USA) can be programmed to move the spot in the X-Y and Z axis directions. A high numerical aperture oil immersion objective (NA = 1.4, X60, Olympus, Tokyo, Japan) was used to tightly focus the laser in LMNPs dispersion. The laser spot diameter is about 700 nm. The imaging system was used for real-time monitoring of the laser processing. Then, the device was rinsed with deionized water for 20 s and dried for 10 min. Finally, silver wires were connected by silver conductive paint to the gold electrodes on both sides to facilitate subsequent performance tests. The actual photographic image of the tested Liquid Metal strain sensor is shown in Figure 1b (right).

A Keithley 2635b (Tektronix, CA, USA) as a source and measurement unit was used to supply power and observe the current variation by applying a constant voltage of 0.1 V. The measurement device was made up of a tensile machine with two claws precisely controlled by a stepping motor. The strain sensor was attached to two measuring claws of the device, which were used to form different forces by adjusting the bending angles of the sensor. We obtain the resistance change using the equation R = V/I. The response was defined as (R − R_0_)/R_0_. During the test, the strain sensor attached to the measuring claw was initially placed horizontally.

## 3. Results and Discussion

### 3.1. Characterization of the Strain Sensor Based on LMNPs

The SEM and EDS mapping images of LMNPs are shown in Figure 1c. The elements of Ga, In, and O were evenly distributed. The XPS spectra of LMNPs before and after laser processing are shown in Figure 1d. It can be seen intuitively that the position of the peaks of the sensing-materials remains almost unchanged before and after laser processing.

**Figure 2:**
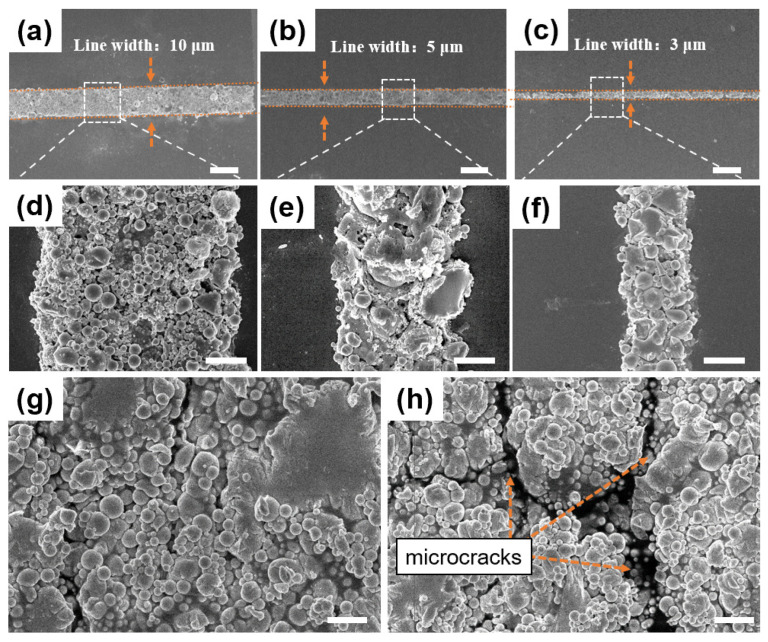
The SEM and enlarged view images with various width of 10 μm (**a**), 5 μm (**b**), and 3 μm (**c**) The scale bar is 10 μm. (**d**–**f**) The enlarge views of squares (**a**–**c**). The scale bar is 2 μm. (**g**) The SEM of the sensor in inward bending states. The scale bar is 1 μm. (**h**) The SEM of the sensor in outward bending states. The scale bar is 1 μm.

The microwires of LMNPs were prepared by adjusting the laser power with a filter. When the laser power was low (<13 mW), the optical force generated by the optical site of the focused beam was small and the optical trap center could not capture more particles, so the processed microwire discontinuous nanoparticles could not be deposited on the substrate surface. When the laser power was excessive (>15 mW), the external oxide shell of the LMNPs cracked and the internal metal leaked. The microwire was completely sintered and conductive, which could not be strain sensing. While the laser processing power was appropriate (13–15 mW), the LMNPs were captured at the laser focus and partially sintered in the laser scanned area forming a continuous and sensing microwire. As shown in Figure 2, the LMNPs are captured, the surface morphology of the microwires prepared using laser processing is complete and continuous with clear edges, and the LMNPs in the microwire are closely packed and arranged in order. According to the set of microwire widths, the microwires with line widths of 3, 5, and 10 μm can be obtained as shown in Figure 2a–f. The high processing accuracy provides a material basis for micro sensing detection. The SEM of the sensor in different strain states (inward and outward) is shown in Figure 2g,h, respectively. When the strain sensor is in the inward bending state, the LMNPs are closely packed. In the outward bending state, some tiny gaps are discovered. This could be the basis for strain sensing.

### 3.2. Strain Sensitivity Investigation of the Strain Sensor Based on LMNPs

**Figure 3:**
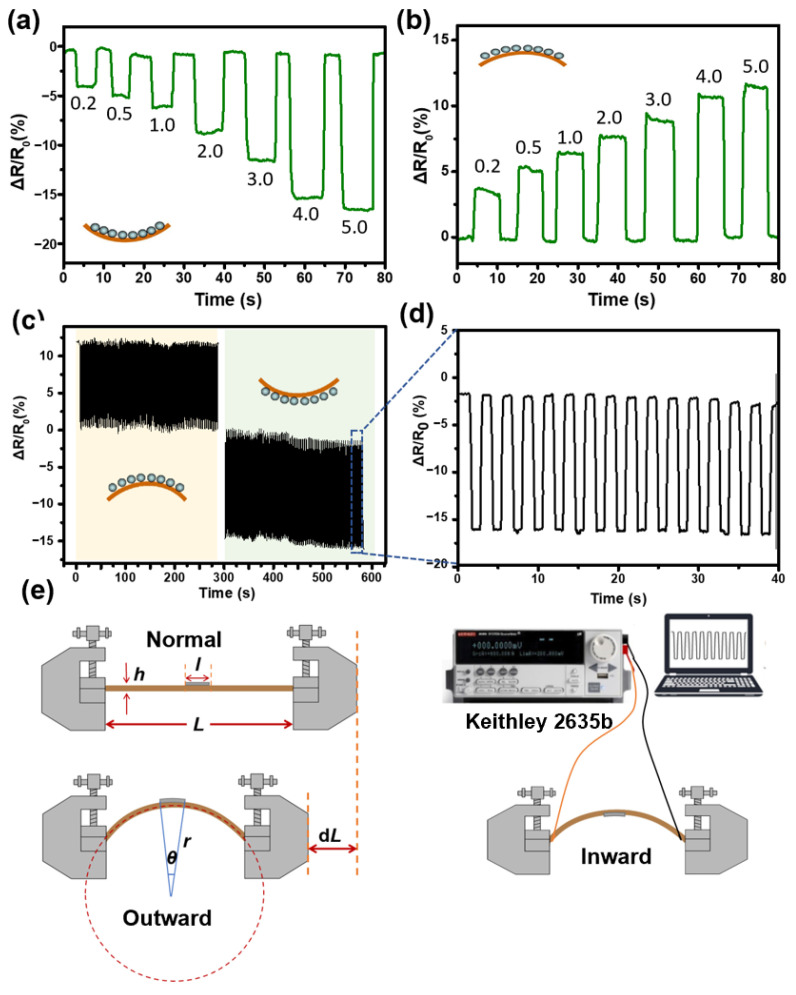
The response of the strain sensor at different dL states (from 0.2 mm to 5.0 mm/10 mm) by applying different inward strains (**a**) and outward strains (**b**). (**c**) The response of 300 continuous cycles by applying a strain of 0.30% in the inward and outward states. (**d**) Enlarged area of (**c**). (**e**) Schematic of measurement setup to measure the performance and the definition of the physical of the strain sensor.

The force sensing properties of the microwire flexible strain sensor based on LMNPs were studied. The sensor was placed between two claws of the tensile machine. By setting the program of the tensile machine to adjust the distance of the two claws, the strain sensor was in different bending states, thus forming different strains. The accuracy of the tensile machine tensile machine is 100 μm, ensuring very small movement errors. The performance parameters of the strain sensor were measured using a digital source meter. A constant voltage of 0.1 V was applied to the microwire strain sensor to detect the current and resistance changes in the microwire sensor in different states. The schematic of measurement setup to measure the performance and the definition of the physical of the strain sensor was shown in Figure 3e. During the test, the strain sensor attached to the measuring claw was initially placed horizontally. The original length of the sensor (*L*) was the distance of the two claws. The thickness of the PI substrate (*h*) was 100 μm.

The relationship between the move distance (*dL*) and the bending radius (r) can be calculated using the following equation [28]:

(1) $$r=\frac{L}{2\pi \sqrt{\left(dL/L\right)-(\pi^{2}h^{2}/12L^{2})}}$$

The mechanical strain was calculated as ε = *h*/2r when the strain sensor was in the outward bending state. Similarly, in the inward bending state, the mechanical strain was calculated as ε = *h*/2r. Combining this with Equation (1):

(2) $$\varepsilon =\pm \frac{\pi h\sqrt{\left(dL/L\right)-(\pi^{2}h^{2}/12L^{2})}}{L}$$

Figure 3a,b showed the relative resistance changed, (R − R_0_)/R_0_, when the strain sensor detected different strains which caused by the changes of the distance of the two claws (*dL*) from 0.2 mm to 5.0 mm/10 mm. The greater the *dL*, the greater the strain. In the inward bending state, it was negative (Figure 3a). On the contrary, in the outward bending state, it changed to be positive (Figure 3b). With the increase in the *dL* and the gradual increase in the strain, the absolute value of the relative resistance of the sensor increased in both the outward and inward bending state. Specifically, intervals between neighboring LMNPs increase when outward strain, increasing the charge transfer distance and therefore the sensor resistance. In contrast, inward strain compresses the nanoparticles, so they overlap each other, shortening the charge transfer distance and decreasing the sensor resistance. This corresponded to the above result in Figure 2g,h. The test results show that the LMNPs strain sensor can effectively sense different strains. The strain sensors show high response even at deformations of 200 μm, indicating excellent ability to monitor fine movements. After the sensor experiences the cycle of applying and releasing strain, the relative resistance returns to the initial value, which indicates good reversibility of the sensor.

The outward or inward bending strain at ε of 0.30% was, respectively, applied to the sensor, and more than 300 cycles of applying and releasing strain were repeated to evaluate the repeatability and durability, which are shown in Figure 3c. The details are seen in Figure 3d. The test results show the current response changes of the sensor are the same in multiple test cycles, showing good consistency with an of error less than 0.1%. Compared with the initial state, the current response change is small after multiple cycles, which indicates that the sensor has excellent repeatability and durability.

**Figure 4:**
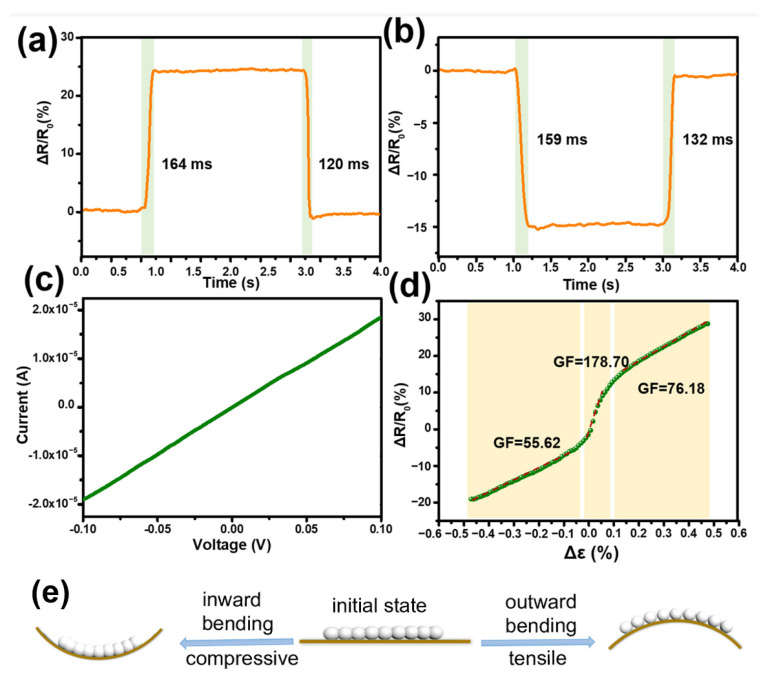
Time response of the microwire-based strain sensor under (**a**) outward and (**b**) inward bending of 0.30%. The green shaded area in the figure shows the response time. (**c**) I–V conductivity measurement of the LMNPs microwire-based strain sensor. (**d**) Relative resistance changes of the sensor under different strains. The GF values were obtained using linear fitting. (**e**) Sensing mechanism of the strain sensor.

The response recovery time of the sensor was detected at outward and inward bending states with ε of 0.30%, as shown in Figure 4a,b, respectively. The response time and recovery time are about 159 ms and 120 ms, which are superior to motion monitoring systems and robotic applications [29]. We then tested the initial state of the strain sensor. The current and voltage curves (I–V curves) were obtained by applying voltages from −0.1 V to 0.1 V. The results are presented in Figure 4c. The I–V characteristics shown demonstrate that the current of the microwire linearly increased with the applied voltage, which proves the existence of an ohmic contact between the electrodes and microwires.

Sensitivity is one of the most important performance parameters of the strain sensor to monitor subtle movements, expressed by the gauge factor (GF), which is defined as GF = (R − R_0_)/R_0_/ε [30]. The GF values were obtained using linear fitting. As shown in Figure 4d, the GF value is 55.62 for strains ranging between −0.47% and −0.042% in the inward bending state. After the strains changed from inward to outward, the GF increased by 1.5 times to 76.18 between 0.012% and 0.047% with linearity of R^2^ 0.999 by linear regression analysis. It is surprising that the GF surges to 178.70 in a relatively small range (−0.0042–0.064%), with the conversion of the strain direction. Although this only exists in a very small range of specific scenarios, it still has a very large significance. The high sensitivity signified that the sensor has the ability to accurately sense subtle mechanical strains. The mass of the reversible contact between the LMNPs, when the strain sensor was subjected to bending, played a significant role in its outstanding sensitivity.

The schematic of the sensing mechanism of the strain sensor is shown in Figure 4e. When the strain sensor senses the inward bending strain (compressive strain), the LMNPs are gradually compressed with the gradual increase in the bending strain. The distance between the LMNPs on both sides of the microcracks gradually decreases, the LMNPs are overlapped and arranged more closely, as in Figure 2g, which shortens the charge transfer distance. As a result, the charge penetration path increased, and the resistance of the strain sensor decreased. When the strain sensor senses the outward bending strain (tensile strain), the initial microcracks in the LMNPs film gradually expand with the increase in the tensile strain, and at the same time, new microcracks are continuously generated; refer to Figure 2h. The contact area between nanoparticles decreases, and the electron transport path becomes narrower and longer. Therefore, the resistance of the strain sensor gradually increases. There are a lot of changeable microcracks in alloy nanoparticle films. In the strain detection range of the sensor, the external strain makes the microcracks in the film expand or be compressed, which significantly affects the transmission distance and path of electrons between particles. Therefore, the relative resistance of the strain sensor changes remarkably. The flexible strain sensor based on LMNPs can accurately sense different tensile and inward compressive strains.

### 3.3. Strain Sensor Based on LMNPs for Human Motion Detection

The microwire flexible strain sensor prepared by FsLDW is very small in size, and the actual sensing area has only 100 square microns. It exhibits high wearing comfort and excellent force sensing performance. To explore the application of the micrometer wire flexible strain sensor, we fixed the sensor on different face and neck parts of the human body using adhesive tape. The signals can be detected and recorded by a source meter connected to the sensor with silver wire in real time when human motion changed.

**Figure 5:**
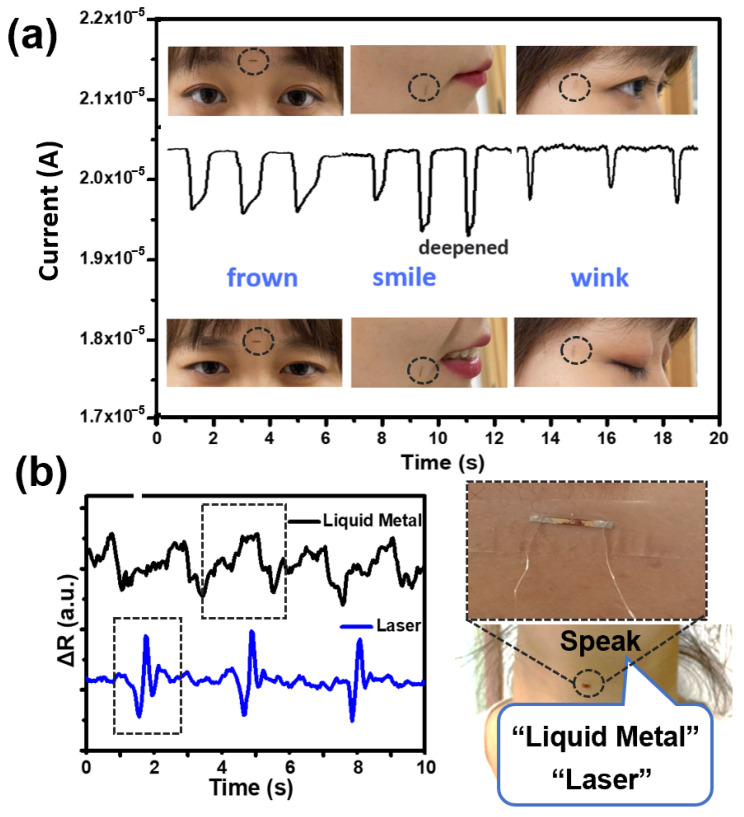
Applications of strain sensor based on LMNPs. (**a**) We fixed the strain sensor on the human face, including the mouth, forehead and eyes. The exact location is shown in the circle. The response of the strain sensor was achieved when the facial expression changed, including frowning, smiling and winking. (**b**) We fixed the strain sensor on the neck to detect vocal cord vibration and the response signal of various words, “liquid metal” and “laser”. One cycle is shown in the box.

We fixed the strain sensor on the human face, including the mouth, eyes and forehead, exact locations are shown at the circle in Figure 5a. The facial expression changes cause the corresponding muscles to be in different motion states. The current change in the sensor was recorded in real time and the test results are shown in Figure 5a. Frowning, smiling, and winking can change the current. Repeating the corresponding action caused the current change in the sensor to also occur repeatedly. The strain sensor can reflect well the change in facial expression and conduct the real-time monitoring of facial expressions. The sensor can clearly measure various vibrations of human vocal cords. We attached the micro linear strain sensor to the throat of the tester, and it avoided the noise impact caused by the outside through the closeness of the microwire sensor to the part of the human body to be detected. The tester spoke different English words and repeated these words three times; the test results in terms of resistance change are shown in Figure 5b using the calculation of R = V/I. Different English words, “laser” and ’liquid metal”, have different waveform characteristics. This indicates that the flexible strain sensor based on liquid metal microwires can detect various vocal cord vibrations and distinguish different words, which can be used for speech recognition.

## 4. Conclusions

In summary, we have developed a high-performance strain sensor for the detection of human motion and subtle strain based on a liquid metal microwire. The small flexible strain sensor has a higher gauge factor (GF = 76.18) for detecting subtle forces accurately and was prepared by FsLDW. The strain sensor also has an excellent short response time of 159 ms and a recovery time of 120 ms. We compared the GF value of this sensor with other reported strain sensors (Table 1), concluding that in addition to having a much easier and cheaper fabrication process, our strain sensor shows superior performance. It is significant for the development of real-time monitoring of human physiological parameters.

## Figures and Tables

**Table 1:** Device performance of strain sensors.

Materials	Methods	GF	Sensing	Ref.
MXene/CNT	Layer-by-layer spraycoating	4.35	0.1–0.6%	[31]
4 nm AuNPs	Layer-by-layer spincoating and contact	14	0–0.12%	[13]
Carbon Black and carboxymethyl cellulose	Dip-coating	4.3	0–0.6%	[13]
AgNW/MoS2	Mix two materials	5.96	0–3	[18]
AuNP thin film	Dip-coating	19.94	0.1–0.5%	[32]
Graphene	3D-printed	10	2–10%	[33]
LMNPs	FsLDW	76.18	0–0.48%	This work

## Data Availability

The data presented in this study are not available due to privacy.
